# Longitudinal Association of Sleep Duration with Depressive Symptoms among Middle-aged and Older Chinese

**DOI:** 10.1038/s41598-017-12182-0

**Published:** 2017-09-18

**Authors:** Yujie Li, Yili Wu, Long Zhai, Tong Wang, Yongye Sun, Dongfeng Zhang

**Affiliations:** 1 Department of Epidemiology and Health Statistics, Medical College, Qingdao University, Qingdao, China; 2 Qingdao Center for Disease Control and Prevention, Qingdao, China; 3 Department of Nutrition and Food Hygiene, Medical College, Qingdao University, Qingdao, China

## Abstract

This study aimed to evaluate the associations of nighttime sleep duration and midday napping with risk of depressive symptoms incidence and persistence among middle-aged and older Chinese. Data from China Health and Retirement Longitudinal Study, CHARLS (2011–2013), were analyzed. Depressive symptoms were identified by the 10-item version of the Centre for Epidemiological Studies Depression scale (CESD-10). Multivariate binary logistic regression models were fitted. There were 7156 individuals with CESD-10 scores < 10 and 3896 individuals with CESD-10 scores ≥ 10 at baseline included in this study. After controlling for potential covariates, nighttime sleep duration <6 hours was associated with high risk of incident depressive symptoms (OR = 1.450, 95%CI: 1.193, 1.764 for middle aged population, and OR = 2.084, 95%CI:1.479, 2.936 for elderly) and persistent depressive symptoms (OR = 1.404, 95%CI: 1.161, 1.699 for middle aged population, and OR = 1.365, 95%CI: 0.979, 1.904 for elderly). For depressed individuals, longer midday napping (≥60 minutes) was associated with lower persistent depressive symptoms (OR = 0.842, 95%CI: 0.717, 0.989). Our study concluded that short nighttime sleep duration was an independent risk factor of depressive symptoms incidence and persistence. Depressed individuals with long midday napping were more likely to achieve reversion than those who have no siesta habit.

## Introduction

Depression is a common psychiatric disorder, affecting an estimated 350 million people worldwide^1^. In China, about 40% of elderly aged ≥60 years had reported depressive symptoms^2^. Depression is associated with decreased physical, cognitive and social function. Moreover, most persons with depression remain untreated, and experience fluctuating symptom levels over time^3^, leading to increased risk of morbidity and suicide^4^. It is predicted that depression would be the second leading cause of disability by 2020^5^. The prevention and treatment of depression have become a hot topic in the field of public health.

Sleep, as a very important health-related factor, has been found to play a role in the development of many diseases^6–8^ and even all-cause mortality^9^. As for the role of night sleep duration in incidence of depression, a recent meta-analysis^10^ of prospective studies concluded that excessively short or long sleep duration was associated with incidence of depression in adults. However, among the 7 studies included in this meta-analysis, 6 studies were carried out in the United States, the seventh, conducted in Japan, had no statistically significant results^11^. Actually, evidences for the longitudinal association of sleep duration with depression among Asians were very limited. In addition, as far as we know, only three previous studies focused on the effect of sleep duration on persistence of depression, and they have drawn conflicting conclusions: two of them indicated that only short sleep was associated with the persistence of depression^12,13^, while the rest one study reported a U-shaped relationship between sleep duration and the persistent depression^14^.

Midday napping is prevalent in China, especially among older adults and is widely accepted as a healthy lifestyle from a cultural perspective^15^. Although accumulative evidence from western populations showed that daytime napping might be a marker of underlying health problems^16^, some experts think that, unlike the nap in the morning or evening, appropriate midday napping, might be restorative and could relieve stress^17,18^. To date, the effect of midday napping on depression has not been explored in-depth.

In this 2-year long longitudinal study, we aimed to investigate whether nighttime sleep duration and midday napping were independently associated with incidence and persistence of depressive symptoms among Chinese aged ≥45 years.

## Methods

### Participants

**Figure 1:**
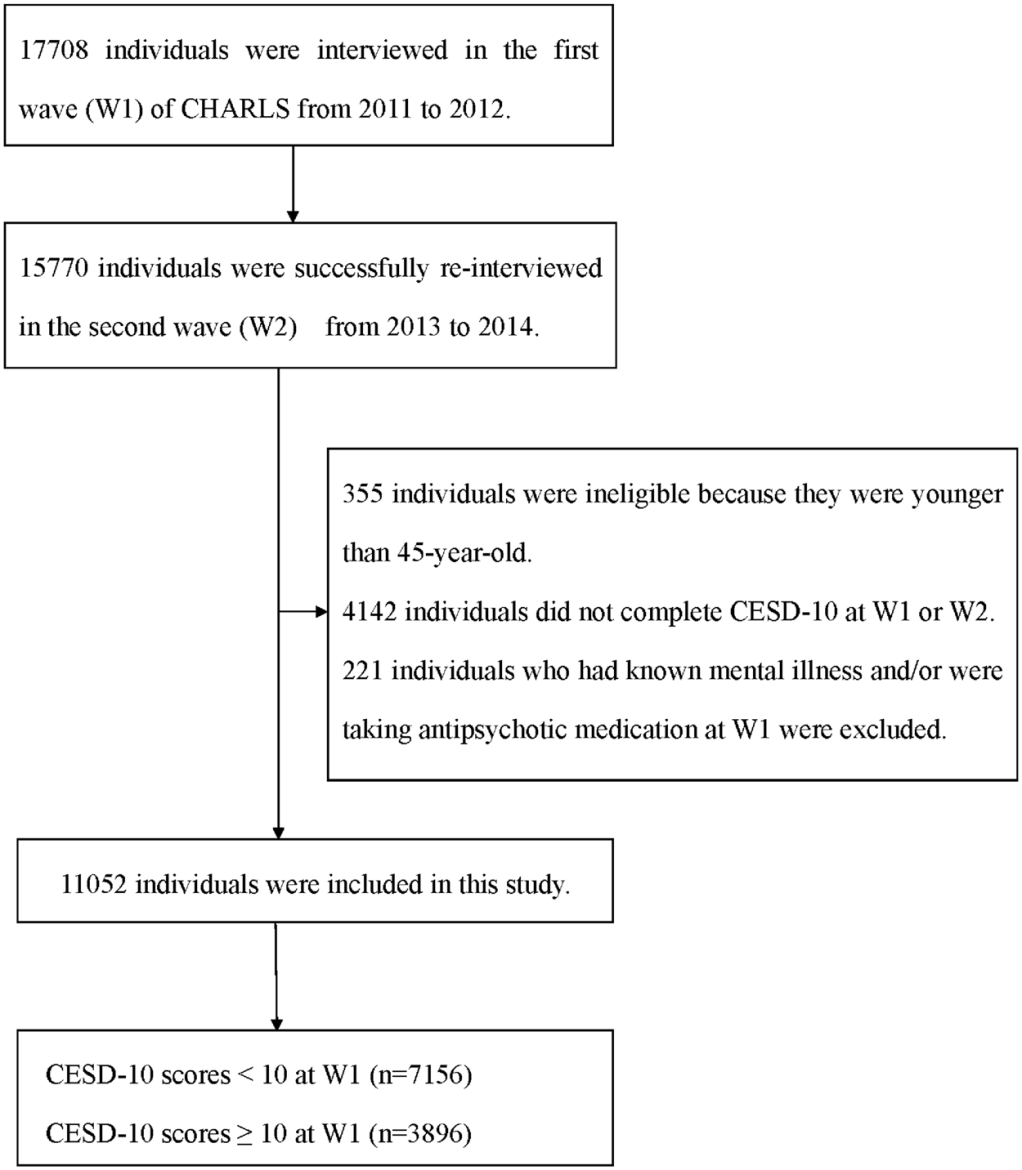
Participants’ flow in the study.

The data analyzed in this study were derived from the China Health and Retirement Longitudinal Study (CHARLS), a nationally representative survey on community-based population aged 45 years or older. The CHARLS was conducted by the National School of Development at Peking University. Details for the procedures of its multistage stratified sampling were described elsewhere^19^. Briefly, the survey included two waves covering 150 county-level units distributed in 28 provinces of China except Tibet. Data for participants’ demographic characteristics, health-related behaviors and lifestyles, health condition, as well as anthropometric and laboratory measurements were collected by well-trained clinicians in a face-to-face, computer-aided personal interview (CAPI) manner. A total of 17,708 individuals agreed to participate in the baseline (wave 1, W1) survey from 2011 to 2012, resulting in a respondent rate of 80%. The second wave (wave 2, W2) survey successfully re-interviewed 15,770 people among them from 2013 to 2014. In the current analyses, 355 individuals who were younger than 45-year-old, 4142 individuals who did not complete depression measurements at W1 or W2, as well as 221 individuals who had known mental illness and/or were taking antipsychotic medication at W1 were excluded. Thus, there were 11,052 individuals included in the final sample (Fig. 1).

### Depressive symptoms

Depressive symptoms were identified by using the 10-item version of the Centre for Epidemiological Studies Depression scale (CESD-10), which had high validity and reliability in Chinese population^20^. The time frame for the CESD-10 questions refers to the week prior to the interview. There were 4 Likert scale answers varying from “rarely or none of the time (<1 day)” to “most or all of the time (5–7 days)” for each item. For the 8 negatively worded items, the answers were coded as 0, 1, 2, 3 scores from “<1 day” to “5–7 days”, while for the 2 positively worded items, the answers were reverse- coded. The sum total is 0–30 and that a total score of ≥10 was defined as clinically significant depressive symptoms.

Among individuals with total CESD-10 score <10 at W1, those who were subsequently diagnosed as having depression by a medical doctor or who endorsed the CESD-10 scores ≥10 at W2 were grouped as incidence of depressive symptoms, and the rest were grouped as non- depressive symptoms. Among individuals with CESD-10 scores ≥10 at W1, those who were taking anti-depressant medicine or CESD-10 scores ≥10 at W2 were grouped as persistent depressive symptoms, and the rest were grouped as reversion of depressive symptoms.

### Sleep duration

Self-reported sleep duration was ascertained with the following question: “During the past month, how many hours of actual sleep did you get at night (average hours for one night)?” and “During the past month, how long did you take a nap after lunch?” Considering the different sleep habits between middle aged and elderly population, a sleep duration of 7–9 hours^21^ per night for middle aged population (45–65 years old) and 7–8 hours^11^ for elderly (≥65 years old) were chosen as the referent categories. Nighttime sleep duration was categorized as <6 hours, 6–7 hours, 7–9 hours and >9 hours for middle aged population, and <6 hours, 6–7 hours, 7–8 hours and >8 hours for elderly. Midday napping was classed as none, <60 minutes and ≥60 minutes. Lack of midday napping (none) was chosen as the ref.^22^.

### Covariates

Our covariates included age, gender, educational level, marital status, body mass index (BMI), cigarette smoking, alcohol drinking and social activity. Educational level was categorized as illiterate (those who had no formal education), literate (those who did not finished primary school but were capable of reading and/or writing), primary education (those who had finished 6 years of education), middle school education and above (those who had finished more than 6 years of education). Marital status was allocated into two categories: single and married, which “single” included “separated”, “divorced”, “widowed”, and “never married”. BMI was derived by taking body weight (in kilogram) divided by height (in meter) squared. Cigarette smoking was classed as ‘never’(those who had never chewed tobacco, smoked a pipe, smoked self-rolled cigarettes, or smoked cigarettes/cigars), ‘former’ (those who had ever smoked, but has totally quit) or ‘current’ (those who still smoked). And alcohol drinking was classed as ‘never’ (those who had never drank alcoholic beverages such as beer, wine, or liquor or had never drank more than once a month), ‘former’ (those who had ever drank more than once a month, but has quit), ‘current’ (those who still drank more than once a month but less than twice a day) or ‘frequent’ (those who drank more than twice a day). Social activity was ascertained with frequencies of 10 items of social activities (e.g. interacting with friends, playing Ma-jong, chess, cards, going to community club, etc.). The answer with “never”, “not regularly”, “almost every week”, or “almost daily”, was coded as 0, 1, 2 or 3 scores. The total social activity score was constructed by summing the responses to each item, with a possible range from 0 to 30.

### Statistical Analysis

Descriptive statistics for baseline characteristics of the participants were presented according to depressive status. The longitudinal associations of participants’ characteristics with depressive symptoms were first tested by using univariate binary logistic regression models. To estimate the independent effect of baseline sleep duration on incidence or persistence of depressive symptoms, three kinds of binary logistic regression models were fitted: model 1, univariate; model 2: adjusted for age and gender; model 3, adjusted for variables in model 2 plus marital status, education, drinking status, smoking status, BMI, social activity. All analyses were carried out using the Statistical Package for the Social Sciences (SPSS), version 18 (SPSS Inc, Chicago, IL, U.S.A.). Statistical significance was defined by setting the type I error rate at α = 0.05 (two-tailed).

## Results

A total of 7156 participants with the CESD-10 scores <10 were classified as free of depressive symptoms at baseline. During the follow-up period, the cumulative incidence of depressive symptoms was 18.6%. Female gender (p < 0.001), lower educational level (p < 0.001), never drinking (p = 0.022), never smoking (p = 0.007), and less social activity (p < 0.001) were associated with higher incidence of depressive symptoms (Table 1).

**Table 1:** Characteristics of 7156 participants with CES-D 10 score <10 at W1.

Independent Variable	Overall n = 7156	Incidence of Depression at W2		p
		no (n = 5823)	yes (n = 1333)	
Age, year (mean ± SD), n = 7156	58.12 ± 8.94	58.14 ± 8.95	58.06 ± 8.91	0.867
Gender (%), n = 7149				
Male (ref.)	3843	3240(84.3)	603(15.7)	—
Female	3306	2578(78.0)	728(22.0)	<0.001
Education (%),n = 7155				
Illiterate	1473	1128(76.6)	345(23.4)	<0.001
Literate	1122	880(78.4)	242(21.6)	<0.001
Primary education	1657	1344(81.1)	313(18.9)	<0.001
Above junior school (ref.)	2903	2471(85.1)	432(14.9)	—
Marital status (%), n = 7156				
Married (ref.)	6556	5351(81.6)	1205(18.4)	—
Single	600	472(78.7)	128(21.3)	0.076
Drinking status (%), n = 6748				
Never	4724	3764(79.7)	960(20.3)	0.025
Former	395	327(82.8)	68(17.2)	0.506
Current	1266	1079(85.2)	187(14.8)	0.757
Frequent (ref.)	363	307(84.6)	56(15.4)	—
Smoking status (%), n = 7154				
Never	4151	3320(80.0)	831(20.0)	0.007
Former	659	562(85.3)	97(14.7)	0.120
Current (ref.)	2344	1939(82.7)	405(17.3)	—
Social activity, score (mean ± SD), n = 7144	1.63 ± 2.18	1.69 ± 2.20	1.40 ± 2.07	<0.001
BMI, kg/m^2^ (mean ± SD), n = 6141	23.85 ± 3.92	23.88 ± 3.84	23.70 ± 4.27	0.192

CESD-10, the 10-item version of the Centre for Epidemiological Studies Depression scale; W1, wave 1; W2, wave 2; SD, standard deviation; BMI, body mass index.

There were 3896 participants with the CESD-10 scores ≥10 at baseline. Of these, 46.1% had reversion of depressive symptoms while the rest had persistent. As shown in Table 2, female gender (p < 0.001), lower educational level (p < 0.001), single marital status (p = 0.049), never drinking (p = 0.003) and never smoking (p < 0.001) were associated with higher risk of persistent depressive symptoms.

**Table 2:** Characteristics of 3896 participants with CES-D 10 score μ ≥ 10 at W1.

Demographics	Overall n = 3896	Persistent Depression at W2		p
		no (n = 1798)	yes (n = 2098)	
Age, year (mean ± SD), n = 3896	59.37 ± 8.98	59.57 ± 9.17	59.20 ± 8.81	0.172
Gender (%), n = 3895				
Male (ref.)	1537	810(52.7)	727(47.3)	—
Female	2358	987(41.9)	1371(58.1)	<0.001
Education (%)				
Illiterate	1233	521(42.3)	712(57.7)	<0.001
Literate	836	336(40.2)	500(59.8)	<0.001
Primary education	908	457(50.3)	451(49.7)	0.318
Above junior school (ref.)	919	484(52.7)	435(47.3)	—
Marital status (%), n = 3896				
Married (ref.)	3318	1553(46.8)	1765(53.2)	—
Single	578	245(42.4)	333(57.6)	0.049
Drinking status (%), n = 3728				
Never	2798	1221(43.6)	1577(56.4)	0.003
Former	277	137(49.5)	140(50.5)	0.183
Current	511	272(53.2)	239(46.8)	0.511
Frequent (ref.)	142	80(56.3)	62(43.7)	—
Smoking status (%), n = 3896				
Never	2513	1090(43.4)	1423(56.6)	<0.001
Past	303	156(51.5)	147(48.5)	0.908
Current (ref.)	1080	552(51.1)	528(48.9)	—
Social activity, score (mean ± SD), n = 3891	1.25 ± 1.85	1.27 ± 1.87	1.24 ± 1.83	0.774
BMI, kg/m^2^ (mean ± SD), n = 3364	23.19 ± 4.00	23.29 ± 3.95	23.10 ± 4.03	0.140

CESD-10, the 10-item version of the Centre for Epidemiological Studies Depression scale; W1, wave 1; W2, wave 2; SD, standard deviation; BMI, body mass index.

Table 3 lists the results of logistic regression analyses for individuals without depressive symptoms at baseline. After adjustment for potential confounders, shorter nighttime sleep duration was independently associated with increased incidence of depressive symptoms in each model. Compared with reference sleep duration, the fully adjusted ORs (95% CI) of nighttime sleep duration <6 h were 1.450 (1.193, 1.764) for middle aged population and 2.084 (1.479, 2.936) for elderly, respectively. No models showed significant effect of longer nighttime sleep on incidence of depressive symptoms. Model 1 suggested that compared with lack of siesta, midday napping ≥60 minutes was associated with lower risk of depressive symptoms incidence (OR = 0.872, 95% CI: 0.762, 0.998). However, after adjustment for more covariates, the association had no statistical significance.

**Table 3:** Longitudinal association of nighttime sleep duration/midday napping with incident depressive symptoms in 7156 participants with CES-D 10 score <10 at W1.

Variables	Model 1	Model 2	Model 3
	OR 95%CI	OR 95%CI	OR 95%CI
**Nighttime Sleep Duration, hour**			
age 45–65 (n = 5505)			
<6	**1.641(1.380,1.951)** ^*^	**1.634(1.373,1.944)** ^*^	**1.450(1.193,1.764)** ^*^
6–7	1.160(0.977,1.377)	1.172(0.986,1.394)	1.205(0.995,1.459)
7–9 (ref.)	1	1	1
>9	1.294(0.910,1.840)	1.275(0.895,1.816)	1.015(0.676,1.52)
age ≥65 (n = 1651)			
<6	**1.903(1.398,2.592)** ^*^	1.922(1.411,2.619)*	**2.084(1.479,2.936)** ^*^
6–7	**1.571(1.134,2.176)** ^*^	1.586(1.144,2.199)*	**1.602(1.115,2.301)** ^*^
7–8 (ref.)	1	1	1
>8	1.497(0.958,2.340)	1.512(0.967,2.364)	1.104(0.639,1.905)
**Midday Napping, minute**			
0 (ref.)	1	1	1
<60	0.925(0.785,1.091)	0.960(0.813,1.133)	0.987(0.821,1.187)
≥60	**0.872(0.762,0.998)** ^*^	0.888(0.776,1.018)	0.925(0.795,1.077)

Model1 unadjusted.

Model 2 adjusted for age, gender.

Model 3 adjusted for Model 2 + marital status, education, drinking status, smoking status, BMI, social activity.

Bold for p ⩽ 0.05.

*for p ⩽ 0.05.

CESD-10, the 10-item version of the Centre for Epidemiological Studies Depression scale; W1, wave 1; OR, odds ratio; BMI, body mass index.

The longitudinal associations between sleep duration and persistent depressive symptoms in logistic regression models are summarized in Table 4. The association between nighttime sleep duration <6 h and persistent depressive symptoms were significant (OR = 1.404, 95%CI: 1.161, 1.699) for middle aged population and suggestive significant (OR = 1.365, 95%CI: 0.979, 1.904) for elderly. Long sleep showed no significant effect on persistent depressive symptoms. Additionally, individuals who reported ≥60 minutes of midday napping were more likely to achieve reversion of depressive symptoms than those who have no midday napping habit (OR = 0.842, 95%CI: 0.717,0.989).

**Table 4:** Longitudinal association of nighttime sleep duration/midday napping with persistent depressive symptoms in 3896 participants with CES-D 10 score ≥10 at W1.

Variables	Model 1	Model 2	Model 3
	OR 95%CI	OR 95%CI	OR 95%CI
**Nighttime Sleep Duration, h**			
age 45–65 (n = 2842)			
<6	**1.450(1.222,1.721)** ^*^	**1.432(1.206,1.702)** ^*^	1.404(1.161,1.699)*
6–7	1.089(0.885,1.340)	1.091(0.886,1.345)	1.056(0.839,1.330)
7–9 (ref.)	1	**1**	**1**
>9	0.822(0.547,1.236)	0.808(0.536,1.219)	0.649(0.404,1.042)
age ≥65 (n = 1054)			
<6	**1.375(1.018,1.856)** ^*^	**1.389(1.025,1.881)** ^*^	1.365(0.979,1.904)
6–7	0.927(0.631,1.362)	0.983(0.665,1.453)	0.875(0.570,1.344)
7–8 (ref.)	1	1	1
>8	0.747(0.448,1.243)	0.765(0.458,1.279)	0.733(0.405,1.325)
**Midday Napping, min**			
0 (ref.)	**1**	**1**	1
<60	0.983(0.821,1.178)	1.012(0.843,1.214)	0.975(0.797,1.193)
≥60	**0.840(0.728,0.970)** ^*^	**0.854(0.739,0.987)** ^*^	**0.842(0.717,0.989)** ^*^

Model1 unadjusted.

Model 2 adjusted for age, gender.

Model 3 adjusted for Model 2 + marital status, education, drinking status, smoking status, BMI, social activity.

Bold for p ⩽ 0.05.

* for p ⩽ 0.05.

CESD-10, the 10-item version of the Centre for Epidemiological Studies Depression scale; W1, wave 1; OR, odds ratio; BMI, body mass index.

## Discussion

The results from this large prospective study, including 11,052 participants, indicate that compared with reference sleep duration, shorter baseline self-reported nighttime sleep duration was associated with higher incidence and persistence of depressive symptoms 2 years later, among middle-aged and older Chinese. Besides, for depressed individuals at baseline, longer midday napping was associated with a lower risk of persistent depressive symptoms.

Our findings about adverse effect of short nighttime sleep on incidence of depressive symptoms were consistent with the previous literature^23^. Similar to 5 analogous studies^11,23–25^, we didn’t find any significant relationship between long sleep duration and incidence of depressive symptoms either. However, after pooling the 5 studies involving 23,663 participants, the meta-analysis^10^ found that long sleep duration was significantly associated with increased risk of depression. The power of a single study was limited possibly because of the small sample size for long sleep duration in community-based population. For example, in our sample, only 284 participants reported nighttime sleep >9 h. Yamada^22,26^ reported the J-curve relation between daytime nap duration and incidence of type 2 diabetes or metabolic syndrome or cardiovascular disease, but linear relationship between daytime nap duration and all-cause mortality. However, there is no previous study specialize on midday napping and depression. We first reported no effect of midday napping on incident depressive symptom.

As far as we know, there have been few studies that have investigated the longitudinal association of nighttime sleep duration with persistence of depression. Different from our conclusion, a 2-year cohort study reported that both short and long sleep duration were important predictors of persistent depressive disorder^14^. Nevertheless, the other two prospective studies found short sleep duration was associated with persistent depression, while long sleep showed no association with depression^12,13^, which were similar to our observation. It is interesting that our results suggested the benefits of long midday napping on reversion of depressive symptoms. However, there is no previous study on the same topic to be reference.

The mechanisms underlying the association between sleep and depression are still not fully understood. Several potential mechanisms exist by which reduced nighttime sleep duration may increase the risk of incident and persistent depressive symptoms. Firstly, physical tiredness and/or psychological fatigue at daytime caused by poor nighttime sleep^27^ may disrupt circadian rhythms and/or cause hormonal alterations, resulting in incidence of depression^28,29^. Secondly, growing evidences suggested that short/long sleep duration was associated with increase of inflammation level, which has been observed in depressed patients compared to non-depressed population. Low-grade chronic inflammation maybe a key biological pathway to link the sleep and depressive symptoms^30,31^. Thirdly, some researchers suggested that short nighttime sleep could be a part of the prodrome of a mental disorder^32^ or a residual symptom of a prior disorder^33^.

A major strength of this study was its large sample size from a prospective cohort, allowing a much greater possibility of reasonable conclusions. As is well-known, the relationship between sleep and depression is bidirectional and complicated. On the one hand, extreme sleep duration is usually considered as a symptom of depression^34,35^. On the other hand, instead of being a symptom, poor sleep may be a risk factor of depression^36^. Longitudinal design can give more information about the direction of causality. Based on this kind of study design, we observed not only the incidence of depressive symptoms in a normal population but also the persistent status in participants with depressive symptoms.

There were some limitations to consider. Firstly, Sleep duration was assessed at W1 over the previous 1 month. Consideration was not given to possible change in sleep duration between W1 and W2. Secondly, the cognitive status of the participants was not determined and the response of the cognitively-impaired patients may not be reliable. Thirdly, there were 27.3% of eligible participants excluded due to incomplete CESD-10 data. Their inclusion, otherwise, could have modified the association between sleep duration and depressive symptoms as found in the present analysis. Fourthly, the lack of comprehensive information on sleep complaints, snore, and other confounders limited our further investigation into the relationship between sleep duration and depressive symptoms. Fifthly, the items related to “cigarette smoking” in the questionnaire did not set a time frame for “current” and “former” that limited the evidence emerging from the study.

## Conclusion

In summary, results from this study indicated that short nighttime sleep duration was an independent risk factor of incident and persistent depressive symptoms. Depressed individuals with long midday napping were more likely to achieve reversion of depression symptoms than those who have no siesta habit.
